# Antibacterial Activity of Polyoxometalates Against *Moraxella catarrhalis*

**DOI:** 10.3389/fchem.2018.00336

**Published:** 2018-08-14

**Authors:** Nadiia I. Gumerova, Emir Al-Sayed, Lukáš Krivosudský, Hana Čipčić-Paljetak, Donatella Verbanac, Annette Rompel

**Affiliations:** ^1^Universität Wien, Fakultät für Chemie, Institut für Biophysikalische Chemie, Wien, Austria; ^2^Center for Translational and Clinical Research, Croatian Center of Excellence for Reproductive and Regenerative Medicine, School of Medicine, University of Zagreb, Zagreb, Croatia

**Keywords:** bioactive polyoxometalates, metal-oxo clusters, Preyssler archetype, Dawson archetype, minimum inhibitory concentration, time-killing analysis, Gram-negative pathogen

## Abstract

The antibacterial activity of 29 different polyoxometalates (POMs) against *Moraxella catarrhalis* was investigated by determination of the minimum inhibitory concentration (MIC). The Preyssler type polyoxotungstate (POT) [NaP_5_W_30_O_110_]^14−^ demonstrates the highest activity against *M. catarrhalis* (MIC = 1 μg/ml) among all tested POMs. Moreover, we show that the Dawson type based anions, [P_2_W_18_O_62_]^6−^, [(P_2_O_7_)Mo_18_O_54_]^4−^, [As_2_Mo_18_O_62_]^6−^, [H_3_P_2_W_15_V_3_O_62_]^6−^, and [AsW_18_O_60_]^7−^ are selective on *M. catarrhalis* (MIC range of 2-8 μg/ml). Among the six tested Keggin type based POTs ([PW_12_O_40_]^3−^, [H_2_PCoW_11_O_40_]^5−^, [H_2_CoTiW_11_O_40_]^6−^, [SiW_10_O_36_]^8−^, [SbW_9_O_33_]^9−^, [AsW_9_O_33_]^9−^), only the mono-substituted [H_2_CoTiW_11_O_40_]^6−^ showed MIC value comparable to those of the Dawson type group. Polyoxovanadates (POVs) and Anderson type POMs were inactive against *M. catarrhalis* within the tested concentration range (1-256 μg/ml). Four Dawson type POMs [P_2_W_18_O_62_]^6−^, [(P_2_O_7_)Mo_18_O_54_]^4−^, [As_2_Mo_18_O_62_]^6−^, [H_3_P_2_W_15_V_3_O_62_]^6−^ and the Preyssler POT [NaP_5_W_30_O_110_]^14−^ showed promising antibacterial activity against *M. catarrhalis* (MICs < 8 μg/ml) and were therefore tested against three additional bacteria, namely *S. aureus, E. faecalis*, and *E. coli*. The most potent antibacterial agent was [NaP_5_W_30_O_110_]^14−^, exhibiting the lowest MIC values of 16 μg/ml against *S. aureus* and 8 μg/ml against *E. faecalis*. The three most active compounds ([NaP_5_W_30_O_110_]^14−^, [P_2_W_18_O_62_]^6−^, and [H_3_P_2_W_15_V_3_O_62_]^6−^) show bacteriostatic effects in killing kinetics study against *M. catarrhalis*. We demonstrate, that POM activity is mainly depending on composition, shape, and size, but in the case of medium-size POTs (charge is more than −12 and number of addenda atoms is not being higher than 22) its activity correlates with the total net charge.

## Introduction

*Moraxella catarrhalis* is a Gram-negative human mucosal pathogen which causes middle ear infections in infants and children and lower respiratory tract infections in adults with chronic pulmonary disease (Karalus and Campagnari, 2000). *M. catarrhalis* is one of the three major causes of otitis media along with *Streptococcus pneumoniae* and *Haemophilus influenzae* (Del Beccaro et al., 1992). Based on culture isolation and serological studies, *M. catarrhalis* has been implicated as a cause of sinusitis in both children and adults. In addition, *M. catarrhalis* occasionally causes severe infections such as septic arthritis, bacteremia, cellulitis, osteomyelitis, endocarditis, and pericarditis (Karalus and Campagnari, 2000). The fact that *M. catarrhalis* was not considered as an important human pathogen until recently has contributed to the limited research aimed to find vaccines for prevention or selective antibiotics for the treatment of respiratory tract infections (Karalus and Campagnari, 2000).

**Figure 1:**
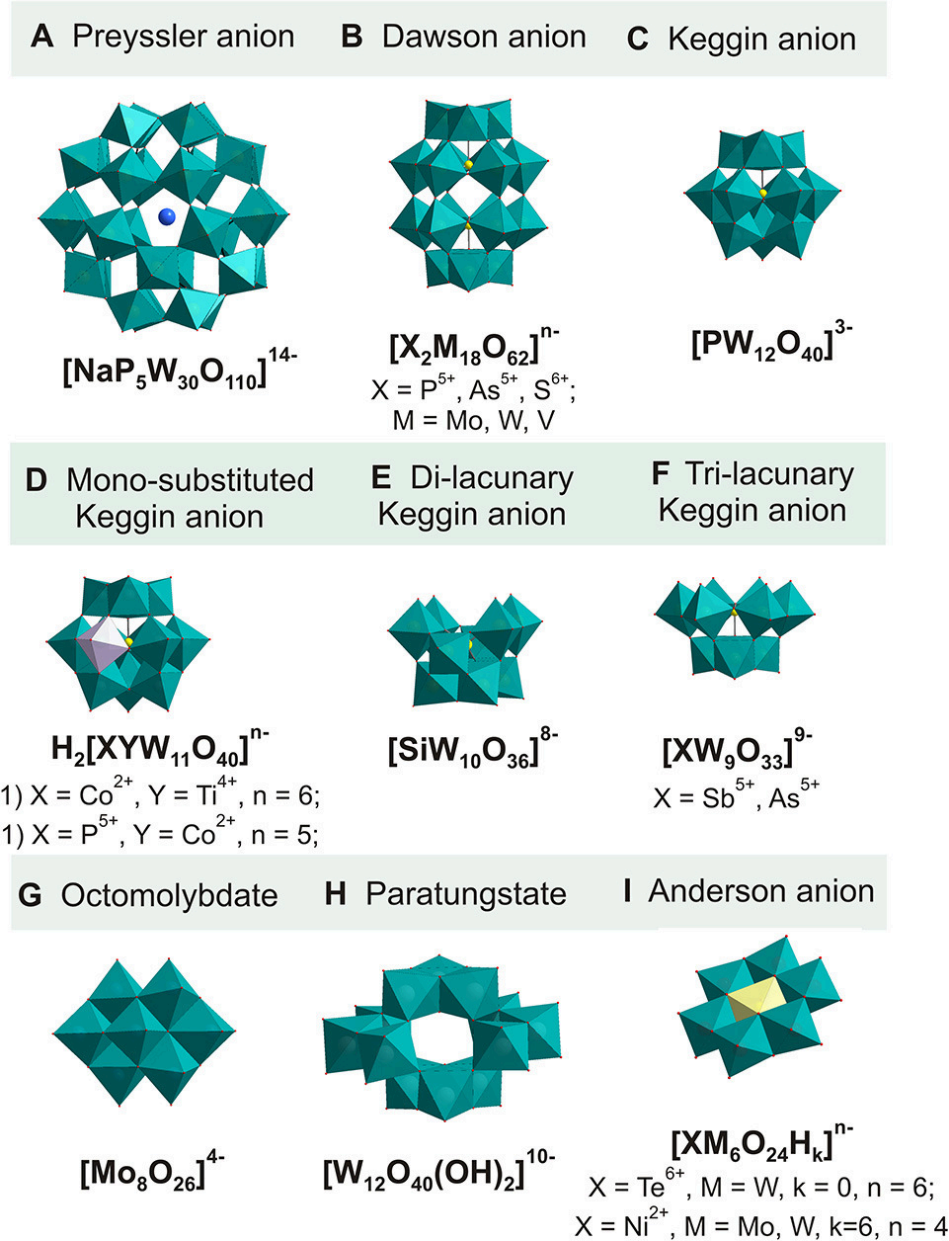
Polyhedral representation of key POM structures tested in this study. **(A)** Preyssler structure **[NaP**_**5**_**W**_**30**_**O**_**110**_**]**^**14−**^ (Jeannin et al., 2007); **(B)** Dawson structure [**X**_**2**_**M**_**18**_**O**_**62**_**]**^n−^ (X = P^5+^, As^5+^, S^6+^; M = Mo, W, V) (e.g., Contant et al., 2007); **(C)** α-Keggin structure **[PW**_**12**_**O**_**40**_**]**^**3−**^ (Phillips, 1950); **(D)** mono-substituted Keggin structure **[H**_**2**_**XYM**_**11**_**O**_**40**_**]**^n−^ [X = Co^2+^, Y = Ti^4+^, *n* = 6 (Kraus et al., 2005); X = P^5+^, Y = Co^2+^, *n* = 5 (Komura et al., 1976)]; **(E)** di-lacunary γ-Keggin structure [**SiW**_**10**_**O**_**36**_**]**^**8−**^ (Téazéa et al., 2007); **(F)** tri-lacunary α-Keggin structure [**XW**_**9**_**O**_**33**_**]**^**9−**^ (X = Sb^5+^, As^5+^) (Tourné et al., 1973); **(G)** octomolybdate **[**α**-Mo**_**8**_**O**_**26**_**]**^**4−**^ (Klemperer, 2007); **(H)** paratungstate **[W**_**12**_**O**_**40**_**(OH)**_**2**_**]**^**10−**^ (Evans and Rollins, 1976); **(I)** Anderson structure [**XM**_**6**_**O**_**24**_**H**_k_**]**^n−^ (X = Te^6+^, M = W, k = 0, *n* = 6 Schmidt et al., 1986; X = Ni^2+^, M = Mo, W, k = 6, *n* = 4 (Rozantsev et al., 2009; Gumerova et al., 2015). Color code: all addenda MO_6_ octahedra, green polyhedra; heteroatom X, yellow spheres or polyhedra; Na in **(A)**, blue sphere; **(C)**, black spheres; CoO_6_ and TiO_6_ in **(D)**, pink polyhedra.

Excessive or improper use of antibiotics led to the development of antibacterial resistance worldwide during the last few decades, suggesting the incidence of these infections may continue to rise. Thus, new active classes of antibiotics are urgently needed for the most common community-acquired respiratory pathogens with emerging antimicrobial resistance. Along with new organic compounds, metal oxides have attracted significant interest over the past decade as they offer alternative modes of antimicrobial action (Dizaj et al., 2014). A particularly attractive sub-class of metal oxides is metal oxide anions, the so-called polyoxometalates (POMs) (Pope, 1983). POMs comprise an array of corner- and edge-sharing pseudo-octahedrally coordinated MO_6_ (M most often V, Nb, Mo, W) units that form an ionic core and are amenable to a variety of chemical transformations (Figure 1). Alongside with applications of POMs in catalysis (Wang and Yang, 2015), nanotechnology (Yamase and Pope, 2006), electrochemistry (Sadakane and Steckhan, 1998), material sciences (Proust et al., 2008), and molecular magnetism (Clemente-Juan et al., 2012), POMs have also been proven to exhibit remarkable biological activity. Due to the highly negative charge, strong acidity, geometry, their use in macromolecular crystallography (Bijelic and Rompel, 2015, 2017; Molitor et al., 2017) and as antimicrobial, (Yamase, 2005; Li et al., 2016; Bijelic et al., 2018), antiviral (Judd et al., 2001), antitumor (Fu et al., 2015), antidiabetes (Nomiya et al., 2001), and antiamyloid-fibril agents (related to Alzheimer's disease) (Gao et al., 2014) has been reported so far and more attention should be given to the biological and therapeutic effect of POMs.

Polyoxotungstates (POTs), polyoxomolybdates (POMos) and polyoxovanadates (POVs) of different structural types have been shown to exhibit synergy with some conventional antibiotics (Yamase et al., 1996; Tajima, 2001) or direct antibacterial activity (Inoue et al., 2005; Bae et al., 2008) against both Gram-negative and Gram-positive bacteria. In general POVs, especially decavanadate, and large, highly negatively charged POMs exhibit a high activity, whereas for example the activity of Keggin type POMs is bacterial strain dependent (Bijelic et al., 2018).

Thus, in this paper, we determined the antibacterial activity of 18 POTs, seven POMos and four POVs. Mainly we focused on two the most common Keggin and Dawson archetypes with different type of addenda atom and number of lacunas. A few examples of isopolytungstates, -molybdates, and -vanadates, as well as Anderson type anions together with larger Preyssler POT were added to the tested group in order to estimate effect of size and charge of anions. The minimum inhibitory concentration (MIC) against *M. catarrhalis* for each POM was determined. The five most active compounds based on Dawson and Preyssler archetypes with MIC < 8 μg/ml were also tested on two Gram-positive organisms *Staphylococcus aureus* and *Enterococcus faecalis* and the Gram-negative bacterium *Escherichia coli*. In addition, time-kill assays were performed against *M. catarrhalis* to study the pharmacodynamics of the POMs of Preyssler and Dawson type with MIC = 1–2 μg/ml by examining the rate of bactericidal activity at varying POM concentrations over time.

## Materials and methods

### Materials

*The **Preyssler** POT* (Figure 1A) (NH_4_)_14_[NaP_5_W_30_O_110_]·30H_2_O (Jeannin et al., 2007); *heteropolymetalates with **Dawson** structure* (Figure 1B): K_6_[P_2_W_18_O_62_]·14H_2_O (Contant et al., 2007), (NH_4_)_6_[P_2_Mo_18_O_62_]·12H_2_O (Briand et al., 2002), [(C_16_H_36_)_4_N]_4_[P_4_Mo_18_O_61_] (Kortz and Pope, 1994), (CH_6_N_3_)_6_[As_2_Mo_18_O_62_]·9H_2_O (Ichida and Sasaki, 1983), [(CH_3_)_4_N]_4_[S_2_Mo_18_O_62_] (Hori and Himeno, 1987), Na_12_[P_2_W_15_O_56_]·25H_2_O (Contant et al., 2007), [N(CH_3_)_4_]_6_[H_3_P_2_W_15_V_3_O_62_]·6H_2_O (Finke et al., 1986), K_6_[As_2_W_18_O_62_]·14H_2_O (Bi et al., 2000), Na_7_[AsW_18_O_60_]·16H_2_O (Jeannin and Martin-Frere, 1979); *heteropolymetalates with **Keggin-based** structures* (Figures 1C-E): Na_3_[PW_12_O_40_]·12H_2_O (Phillips, 1950), (NH_4_)_5_[H_2_PCoW_11_O_40_] (Komura et al., 1976), K_6_H_2_[CoTiW_11_O_40_]·13H_2_O (Kraus et al., 2005), K_8_[SiW_10_O_36_]·10H_2_O (Téazéa et al., 2007), Na_9_[SbW_9_O_33_]·19.5H_2_O (Tourné et al., 1973); Na_9_[AsW_9_O_33_]·19.5H_2_O (Tourné et al., 1973), K_14_[As_2_W_19_O_67_(H_2_O)] (Kortz et al., 2001); K_9_(C_2_H_8_N)_5_[H_10_Se_2_W_29_O_103_]·30H_2_O (Gao et al., 2013); ***isopolymetalates*** (Figures 1F,G): Na_10_[W_12_O_40_(OH)_2_]·20H_2_O (Evans and Rollins, 1976), Na_12_[H_4_W_22_O_74_]·31H_2_O (Miras et al., 2008), [(C_4_H_9_)_4_N]_2_[Mo_6_O_19_] (Klemperer, 2007), [(C_4_H_9_)_4_N]_4_[α-Mo_8_O_26_] (Klemperer, 2007), K_4_Na_2_[V_10_O_28_]·10H_2_O (Lee and Joo, 2003), K_4_[V_4_O_8_(L-tart)_2_]·8H_2_O, tart = C_4_H_2_${\text{O}}_{6}^{4-}$ (Schwendt et al., 2007), K_4_[V_4_O_8_(D-tart)_2_]·8H_2_O, tart = C_4_H_2_${\text{O}}_{6}^{4-}$ (Schwendt et al., 2007); *heteropolymetalates with **Anderson** structure* (Figure 1I)*:* Na_6_[TeW_6_O_24_]·22H_2_O (Schmidt et al., 1986), Na_4_[Ni(OH)_6_W_6_O_18_]·16H_2_O (Rozantsev et al., 2009), Na_4_[Ni(OH)_6_Mo_6_O_18_]·16H_2_O (Gumerova et al., 2015), Na_2_[N(CH_3_)_4_]_2_[Ni(OH)_3_W_6_O_18_(OCH_2_)_3_CCH_2_OH]·9H_2_O (Gumerova et al., 2016) were synthesized according to procedures published elsewhere. Table 1 lists the POMs tested in this study together with notation of their abbreviation. ***Characterization of POMs***. Compounds were identified by IR measurements on a Bruker Vertex 70 IR Spectrometer equipped with a single-reflection diamond-ATR unit. In case of mono-crystalline sample POMs ((NH_4_)_14_[NaP_5_W_30_O_110_]·30H_2_O, K_6_[P_2_W_18_O_62_]·14H_2_O, K_6_H_2_[CoTiW_11_O_40_]·13H_2_O, [N(CH_3_)_4_]_6_[H_3_P_2_W_15_V_3_O_62_]·6H_2_O, Na_10_[W_12_O_40_(OH)_2_]·20H_2_O, Na_12_[H_4_W_22_O_74_]·31H_2_O, Na_6_[TeW_6_O_24_]·22H_2_O, Na_4_[Ni(OH)_6_W_6_O_18_]·16H_2_O, Na_4_[Ni(OH)_6_Mo_6_O_18_]·16H_2_O) were also identified by checking unit cell on a Bruker D8 Venture equipped with multilayer monochromator, MoKα INCOATEC micro focus sealed tube and Kryoflex cooling device.

**Table 1:** Minimum inhibitory concentration (MIC) of POMs against the *M. catarrhalis* (ATCC 23246).

**POM**	**Abbreviation**	**Charge number (z)**	z/m^*^	**MIC, μg/ml**
**HETEROPOLYANIONS**				
	**Preyssler POT (Figure 1A)**			
(NH_4_)_14_[NaP_5_W_30_O_110_]·30H_2_O	**P**_**5**_${\text{W}}_{30}^{14-}$	−14	0.47	1
	**Dawson-based (Figure 1B)**			
K_6_[P_2_W_18_O_62_]·14H_2_O	**P**_**2**_${\text{W}}_{18}^{6-}$	−6	0.33	2
[N(CH_3_)_4_]_6_[H_3_P_2_W_15_V_3_O_62_]·6H_2_O	**P**_**2**_**W**_**15**_${\text{V}}_{3}^{6-}$	−6	0.33	2
[(C_16_H_36_)_4_N]_4_[P_4_Mo_18_O_61_]	**P**_**2**_**O**_**7**_${\text{Mo}}_{18}^{4-}$	−4	0.22	4
(CH_6_N_3_)_6_[As_2_Mo_18_O_62_]·9H_2_O	**As**_**2**_${\text{Mo}}_{18}^{6-}$	−6	0.33	4
Na_7_[AsW_18_O_60_]·16H_2_O	${\text{AsW}}_{18}^{7-}$	−7	0.39	8
(NH_4_)_6_[P_2_Mo_18_O_62_]·12H_2_O	**P**_**2**_${\text{Mo}}_{18}^{6-}$	−6	0.33	>256
[(CH_3_)_4_N]_4_[S_2_Mo_18_O_62_]	**S**_**2**_${\text{Mo}}_{18}^{4-}$	−4	0.22	>256
Na_12_[P_2_W_15_O_56_]·25H_2_O	**P**_**2**_${\text{W}}_{15}^{12-}$	−12	0.8	>256
K_6_[As_2_W_18_O_62_]·14H_2_O	**As**_**2**_${\text{W}}_{18}^{6-}$	−6	0.33	>256
	**Keggin-based (Figures 1C-F)**			
K_6_H_2_[CoTiW_11_O_40_]·13H_2_O	${\text{CoTiW}}_{11}^{6-}$	−6	0.45	16
K_8_[SiW_10_O_36_]·10H_2_O	${\text{SiW}}_{10}^{8-}$	−8	0.8	32
Na_3_[PW_12_O_40_]·12H_2_O	${\text{PW}}_{12}^{3-}$	−3	0.25	128
Na_9_[SbW_9_O_33_]·19.5H_2_O	${\text{SbW}}_{9}^{9-}$	−9	1	256
Na_9_[AsW_9_O_33_]·19.5H_2_O	${\text{AsW}}_{9}^{9-}$	−9	1	>256
(NH_4_)_5_[H_2_PCoW_11_O_40_]	${\text{PCoW}}_{11}^{5-}$	−5	0.45	>256
	**POTs based on lacunary Keggin units**			
K_14_[As_2_W_19_O_67_(H_2_O)]	**As**_**2**_${\text{W}}_{19}^{14-}$	−14	0.74	64
K_9_(C_2_H_8_N)_5_[H_10_Se_2_W_29_O_103_]·30H_2_O	**Se**_**2**_${\text{W}}_{29}^{14-}$	−14	0.48	64
	**Anderson-based (Figure 1I)**			
Na_6_[TeW_6_O_24_]·22H_2_O	${\text{TeW}}_{6}^{6-}$	−6	1	>256
Na_4_[Ni(OH)_6_W_6_O_18_]·16H_2_O	${\text{NiW}}_{6}^{4-}$	−4	0.67	>256
Na_4_[Ni(OH)_6_Mo_6_O_18_]·16H_2_O	${\text{NiMo}}_{6}^{4-}$	−4	0.67	>256
Na_2_[N(CH_3_)_4_]_2_[Ni(OH)_3_W_6_O_18_(OCH_2_)_3_CCH_2_OH]·9H_2_O	**NiW**_**6**_**penta**^**4−**^	−4	0.67	>256
**ISOPOLYANIONS (FIGURES 1G,H)**				
[(C_4_H_9_)_4_N]_4_[α-Mo_8_O_26_]	${\text{Mo}}_{8}^{4-}$	−4	0.5	32
Na_10_[W_12_O_40_(OH)_2_]·27H_2_O	${\text{W}}_{12}^{10-}$	−10	0.45	64
Na_12_[H_4_W_22_O_74_]·31H_2_O	${\text{W}}_{22}^{12-}$	−12	0.54	128
[(C_4_H_9_)_4_N]_2_[Mo_6_O_19_]	${\text{Mo}}_{6}^{2-}$	−2	0.33	>256
K_4_Na_2_[V_10_O_28_]·10H_2_O	${\text{V}}_{10}^{6-}$	−6	0.6	>256
K_4_[V_4_O_8_(L-tart)_2_]·8H_2_O, tart = C_4_H_2_${\text{O}}_{6}^{4-}$	**V**_**4**_**-L-tart**^**4−**^	−4	1	256
K_4_[V_4_O_8_(D-tart)_2_]·8H_2_O, tart = C_4_H_2_${\text{O}}_{6}^{4-}$	**V**_**4**_**-D-tart**^**4−**^	−4	1	>256
**POSITIVE CONTROL**				
Azithromycin (Lode et al., 1996)				0.06

*POMs combined in groups according to their structural type and within the group listed from higher to lower activity*.

* *m - number of addenda atoms*.

### MIC determination

Minimum inhibitory concentrations (MICs) were determined by the broth microdilution method according to guidelines of the Clinical Laboratory Standards Institute (Wikler, 2009). Double dilutions of tested compounds in 96-well microtiter plates were prepared in the concentration range of 1-256 μg/mL. *E. coli* (ECM1556) and *S. aureus* (ATCC 29213) were grown on Mueller-Hinton agar plates (by Becton Dickinson, USA), whereas *E. faecalis* (ATCC29212) and *M. catarrhalis* (ATCC 23246) were grown on Columbia agar with 5% defibrinated sheep blood. Inocula were prepared by direct colony suspension method and plates were inoculated with 5·10^−4^ CFU/well. Results were determined by visual inspection after 20–22 h of incubation at 37°C in ambient air. Testing was performed by the standard broth microdilution method with azithromycin (Lode et al., 1996) as the reference antibiotic to assess test validity.

### Time-killing assay

*M. catarrhalis* inoculum was prepared by direct colony suspension in sterile saline and the organism density was matched to 1.0 McFarland turbidity standard. The bacterial suspension was further diluted in cation-adjusted Mueller-Hinton Broth in 1:50 ratio to obtain the starting inoculum of 5·10^5^–5·10^6^ colony-forming units (CFU)/mL. Tested POMs were added to tubes containing 6 mL of bacterial suspension, in concentrations corresponding to 1×, 5×, and 10×MIC, while the control antibiotic azithromycin was tested with 1× and 10×MIC. One tube was used as a drug-free control. After addition of the POMs, tubes were incubated at 37°C for 24 h. Viable colony counts were determined at 0, 2, 4, 6, and 24 h. At each time-point, a 100 μL aliquot was removed from each tube and 10-fold dilutions were prepared in saline, plated on Columbia agar with 5% defibrinated sheep blood in 20 μL aliquots and incubated on 37°C for 24 h. The lower limit for quantifying colony counts was 200 CFU/mL. Bactericidal activity was defined as a ≥3 log_10_ reduction in CFU/mL (Barry et al., 1999).

## Results and discussion

### Antibacterial activity of preyssler and dawson type POTs and POMos

The antibacterial activity of the 29 POMs against the Gram-negative *M. catarrhalis* was evaluated by means of MIC (Table 1). The highest activity with a MIC range of 1-8 μg/ml was observed for POMs with Preyssler type (Figure 1A) and Dawson (Figure 1B) structure.

Moreover, the most active POM on *M. catarrhalis*, namely the Preyssler anion **P**_**5**_${\text{W}}_{30}^{14-}$ (Figure 1A) (MIC = 1 μg/ml), was additionally tested on the Gram-positive organisms *S. aureus* and *E. faecalis* and the Gram-negative *E. coli*, which are major human pathogens that cause a wide range of clinical infections (Table 2). **P**_**5**_${\text{W}}_{30}^{14-}$ exhibited good activity against *S. aureus* with MIC = 16 μg/ml and *E. faecalis* with MIC = 8 μg/ml, which is the same as for the clinically applied drug azithromycin (Lode et al., 1996), however, it performed inactive against the Gram-negative *E. coli*. The chitosan-**P**_**5**_**W**_**30**_ nanoassembly has already demonstrated high anticancer activity, which is considered to arise due to high number of phosphorous and tungsten atoms (Shah et al., 2014). Remarkably, the Se-containing lacunary anion **Se**_**2**_${\text{W}}_{29}^{14-}$, which is of comparable size and equally charged, exhibited significantly lower MICs (64 μg/ml). This indicates the importance and the influence of the structure, shape, and composition for the antibacterial activity, justifying more detailed studies to elucidate the structure-activity relationship.

**Table 2:** Minimum inhibitory concentration (MIC) of Dawson and Preyssler type POMs against the *M. catarrhalis* strains.

**Compound**	**MIC, μg/ml**		
	***S. aureus* (ATCC 29213)**	***E. faecalis* (ATCC29212)**	***E. coli* (ECM1556)**
**PREYSSLER ANION**			
**P**_**5**_${\text{W}}_{30}^{14-}$	16	8	>256
**DAWSON-BASED ANIONS**			
**P**_**2**_**O**_**7**_${\text{Mo}}_{18}^{4-}$	>256	>256	>256
**As**_**2**_${\text{Mo}}_{18}^{6-}$	256	>256	>256
**P**_**2**_**W**_**15**_${\text{V}}_{3}^{6-}$	>256	>256	>256
${\text{AsW}}_{18}^{7-}$	>256	>256	>256
Azithromycin (positive control)^*^	1	8	0.25

* *MICs for azithromycin were obtained in this study*.

**Figure 2:**
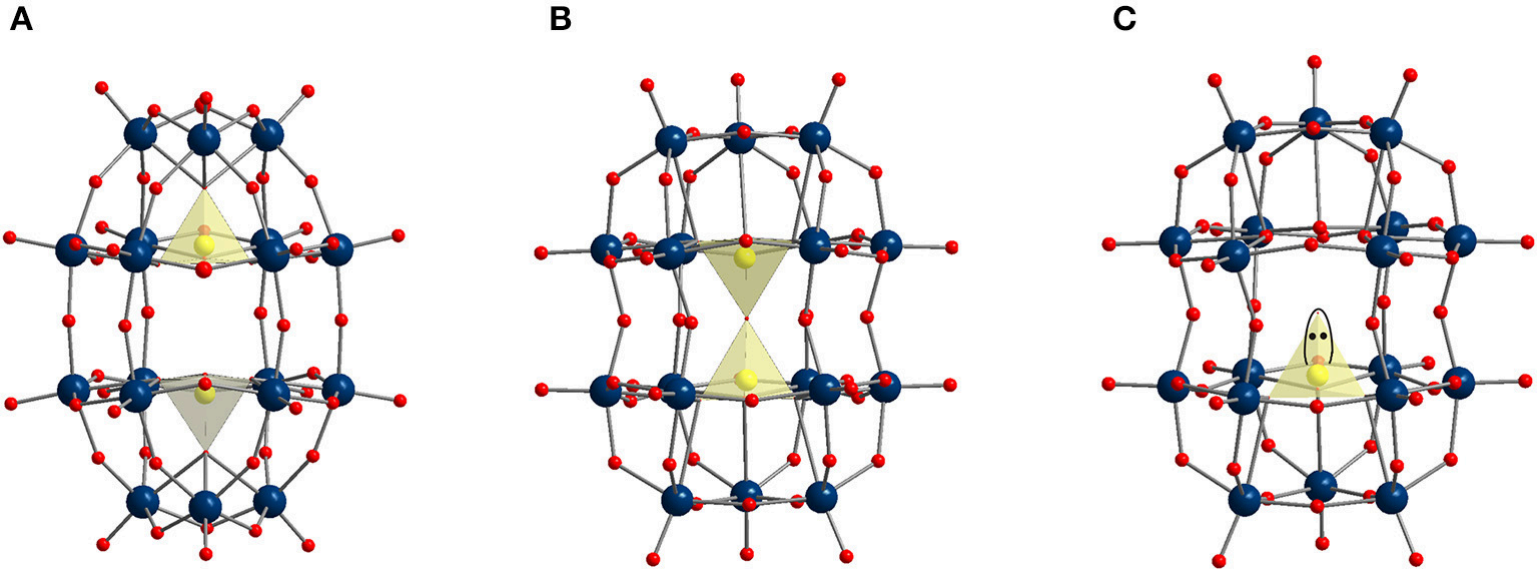
Ball and stick representation of **(A)** classical Dawson type anion **P**_**2**_${\text{Mo}}_{18}^{6-}$ (Briand et al., 2002); **(B)** anion in **P**_**2**_**O**_**7**_${\text{Mo}}_{18}^{4-}$ (Kortz and Pope, 1994); **(C)** anion in ${\text{AsW}}_{18}^{7-}$ (Jeannin and Martin-Frere, 1979). Color code: addenda atom Mo **(B)** or W **(C)**, dark blue spheres; heteroatom P **(B)** or As **(C)**, yellow spheres; O, red spheres.

Except for **P**_**2**_${\text{Mo}}_{18}^{6-}$, **S**_**2**_${\text{Mo}}_{18}^{4-}$, and **As**_**2**_${\text{W}}_{18}^{6-}$, all Dawson type POMs (Figure 1B) tested in this study exhibited potential antibacterial activity exhibiting a MIC within the range of 2–8 μg/ml. Among the Dawson type group, **P**_**2**_${\text{W}}_{18}^{6-}$ and its triple-protonated equally charged vanadium-substituted analog **P**_**2**_**W**_**15**_${\text{V}}_{3}^{6-}$ (Figure 1B) have proven to be the most promising with a MIC of 2 μg/ml suggesting that VO_6_ sites in Dawson type mixed polyoxovanadatotungstates (POVTs) lattice do not have any significant impact on the antibacterial activity, which was observed earlier for Keggin POVTs as they were remarkably more active against *S. pneumoniae* than their corresponding POTs (Fukuda and Yamase, 1997). In the Dawson pair **P**_**2**_${\text{W}}_{18}^{6-}$ (MIC = 2 μg/ml) and **P**_**2**_${\text{Mo}}_{18}^{6-}$ (MIC > 256 μg/ml), the POMo is considered as inactive, whereas for **As**_**2**_${\text{W}}_{18}^{6-}$ (MIC > 256 μg/ml) and **As**_**2**_${\text{Mo}}_{18}^{6-}$ (MIC = 4 μg/ml) the opposite effect is observed. Dawson related compounds, namely ${\text{AsW}}_{18}^{7-}$ (Figure 2C), with one tricoordinated As^III^O_3_ unit (Jeannin and Martin-Frere, 1979), and **P**_**2**_**O**_**7**_${\text{Mo}}_{18}^{4-}$ (Figure 2B), which has a pyrophosphate anion enclosed (Kortz and Pope, 1994), demonstrated higher activity against bacteria (MIC values are 8 and 4 μg/ml, respectively) than classical **As**_**2**_${\text{W}}_{18}^{6-}$ and **P**_**2**_${\text{Mo}}_{18}^{6-}$ (Figure 2A). The presence of highly bioactive and toxic arsenic trioxide in the first case should play a significant role, but difference in the coordination of the heteroatoms in both cases leads to a change of the “rugby-ball-shaped” (Figure 2A) Dawson structure to a “hour-glass” shaped anion (Figures 2B,C), which also may be related to discrepancies in antibacterial activity. These anomalies in the activity of isostructural POTs and POMos indicate that both the hetero- and addenda atoms play a significant role in the bioactivity and that the appropriate combination of these atoms must be decisive for the antibacterial activity.

The superiority of the Dawson structure among four different structural groups of polyoxomolybdates in the inhibition of a tartrate-resistant acid phosphatase (ACP) from *Leishmania donovani* and the tartrate-sensitive ACP from human seminal fluid (prostatic ACP) has been reported previously (Saha et al., 1991). **As**_**2**_${\text{Mo}}_{18}^{6-}$ was the most potent inhibitor and exhibited the highest degree of selectivity against both ACPs. Here, **As**_**2**_${\text{Mo}}_{18}^{6-}$is proved to be a potent antibacterial agent with the third lowest MIC value of 4 μg/ml against *M. catarrhalis*.

### Antibacterial activity of keggin- and anderson based type POTs

Keggin type POTs are known to exhibit antibacterial activities, for example, by increasing the susceptibility of certain bacteria strains toward β-lactam antibiotics (Yamase et al., 1996). In this study the strongest activity was shown for the Keggin based ${\text{CoTiW}}_{11}^{6-}$ (Figure 1D) exhibiting a MIC value of 16 μg/ml. Interestingly, despite consisting of the same isomer of Keggin unit, the classical ${\text{PW}}_{12}^{3-}$ (Figure 1C) and the two mono-substituted ${\text{PCoW}}_{11}^{5-}$ and ${\text{CoTiW}}_{11}^{6-}$ (Figure 1D) showed completely different activities. The most negatively charged ${\text{CoTiW}}_{11}^{6-}$ is the most active compound; however, the charge dependency is not observed in the case of the other two Keggin anions-${\text{PW}}_{12}^{3-}$ with a total charge of −3 exhibited a MIC of 128 μg/ml and ${\text{PCoW}}_{11}^{5-}$ with a total charge of −5 exhibited a value >256 μg/ml. Thus, we assume a decisive role for the accessible TiO_6_ unit in ${\text{CoTiW}}_{11}^{6-}$ (Figure 1D) in the activity against *M. catarrhalis*. ${\text{CoTiW}}_{11}^{6-}$ was previously also shown as the most potent NTPDase inhibitor among six different POTs, (Müller et al., 2006).

The dilacunary ${\text{SiW}}_{10}^{8-}$ (Figure 1E) showed much higher activity than the trilacunary anions ${\text{SbW}}_{9}^{9-}$ and ${\text{AsW}}_{9}^{9-}$ (Figure 1F; 32 μg/ml for ${\text{SiW}}_{10}^{8-}$ against >256 μg/ml for ${\text{SbW}}_{9}^{9-}$ and ${\text{AsW}}_{9}^{9-}$). Nevertheless, the Keggin and Dawson **P**_**2**_${\text{W}}_{15}^{12-}$ lacunary anions did not meet the expectation that more negatively charged compounds exhibit higher antibacterial activity.

The inorganic and organically functionalized Anderson type POTs and POMos (Figure 1I) are inactive against *M. catarrhalis* (Table 1). The inactivity of this type of POM was previously observed for *Helicobacter pylori*, which as well as *M. catarrhalis* is most sensitive to larger POMs (Yamase et al., 1996). It is tempting to speculate that the combination of compact size and small charge of Anderson type anion (Blazevic and Rompel, 2016) is the reason of its antibacterial inactivity.

### Antibacterial activity of isopolymetalates

Among the investigated isopolyanions only two POTs (${\text{W}}_{12}^{10-}$ (Figure 1H) and ${\text{W}}_{22}^{12-}$) and octamolybdate ${\text{Mo}}_{8}^{4-}$ (Figure 1G) showed a MIC value >256 μg/ml. It should be noted, that decavanadate tested in this study (${\text{V}}_{10}^{6-}$) did not show antibacterial activity (MIC >256 μg/ml), which confirms the selective activity of the most common vanadates ${\text{V}}_{10}^{6-}$ and V_4_${\text{O}}_{12}^{4-}$ against *Streptococcus pneumoniae* with MIC values in the range of 4–32 μg/ml (positive control with conventional antibiotics: 2–32 μg/ml; Fukuda and Yamase, 1997). We also included tetranuclear vanadium tartrates (**V**_**4**_**-L-tart**^**4−**^ and **V**_**4**_**-D-tart**^**4−**^) in our study as they, similarly to ${\text{V}}_{10}^{6-}$, are one of the few vanadate species with proved stability and hydrolytic immunity in aqueous solutions over time (Schwendt et al., 2007). However, both POVs were inactive toward *M. catarrhalis*.

### The relationship between the composition of POMs and its activity against *M. catarrhalis*

By analyzing the data in Table 1, it becomes clear that POMs despite having the same or very close charge and size can demonstrate absolutely different activities (e.g. compare Dawson-based **P**_**2**_${\text{W}}_{18}^{6-}$ and **P**_**2**_${\text{Mo}}_{18}^{6-}$ or Keggin-based ${\text{PCoW}}_{11}^{5-}$ and ${\text{CoTiW}}_{11}^{6-}$). As already noted above, there are at least three factors affecting the antibacterial activity: size, charge, chemical composition, and their combination. In order to understand the structure-activity-relationship (SAR) we minimized the influence of one of these factors and compared the main characteristics for phosphorus-containing Keggin ${\text{PW}}_{12}^{3-}$ (Figure 1C), Dawson **P**_**2**_${\text{W}}_{18}^{6-}$ (Figure 1B), and Preyssler **P**_**5**_${\text{W}}_{30}^{14-}$ (Figure 1A) POTs (Table 3). Leastways for these fully saturated (not lacunary) POTs with the same heteroatom ${\text{PW}}_{12}^{3-}$, **P**_**2**_${\text{W}}_{18}^{6-}$, and **P**_**5**_${\text{W}}_{30}^{14-}$ there is a clear dependence in the increase in antibacterial activity with an increase in charge and size and no correlation with respect to the redox potential.

**Table 3:** Dimension and redox characteristics for phosphorus-containing Keggin, Dawson, and Preyssler POTs.

**POT**	**Charge number (*z*)**	Volume/10^−22^ cm^3^^**^	Volume charge density/cm^−3^^**^	*z*/m^*^	Reduction potential, V^**^	**MIC, μg/ml**
**P**_**5**_${\text{W}}_{30}^{14-}$ (Preyssler)	−14	18.48	1,213	0.47	−0.43	1
**P**_**2**_${\text{W}}_{18}^{6-}$ (Dawson)	−6	9.995	961.8	0.33	+0.06	2
${\text{PW}}_{12}^{3-}$ (Keggin)	−3	6.234	771.0	0.25	−0.023	128

* *m-number of addenda atoms*.

** *were taken from López et al. (2006)*.

**Figure 3:**
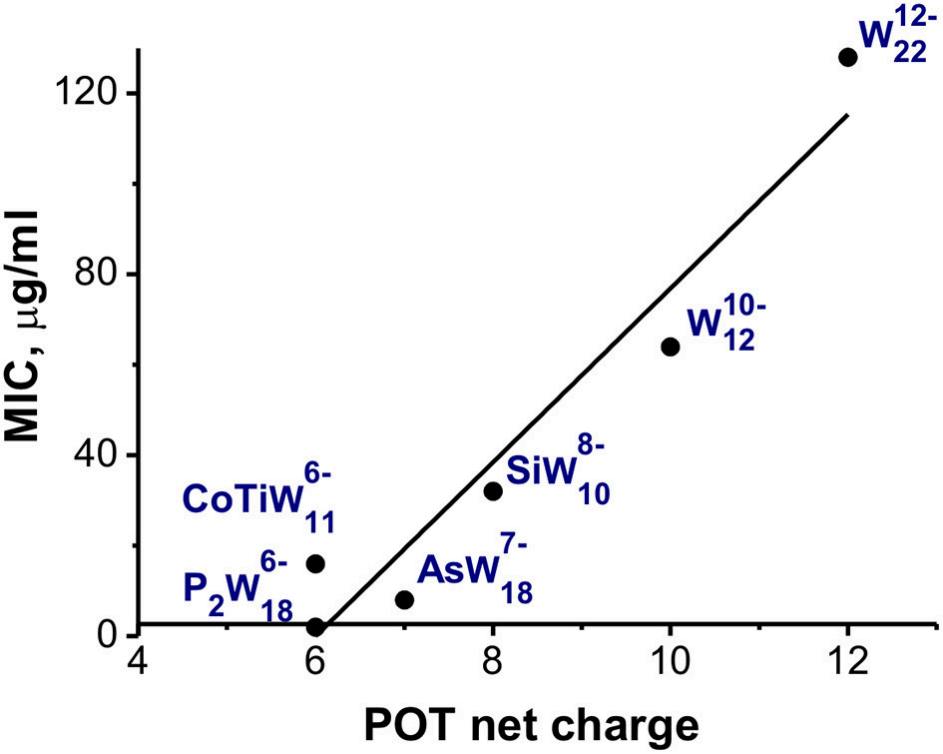
Charge-activity-relationship of POTs against *M. catarrhalis*. POTs **P**_**2**_${\text{W}}_{18}^{6-}$ (Figure 1B) and ${\text{AsW}}_{18}^{7-}$ (Figure 2C) demonstrate Dawson structure, ${\text{CoTiW}}_{11}^{6-}$ (Figure 1D) and ${\text{SiW}}_{10}^{8-}$ (Figure 1E) are Keggin-based anions, whereas ${\text{W}}_{12}^{10-}$ (Figure 1H) and ${\text{W}}_{22}^{12-}$ - isopolytungstates (see Table 1).

No simple SAR was found for all tested POMs, however, narrowing the data set only to the largest tested group, namely POTs with a charge <–12 and with a number of addenda atoms not being higher than 22 it became possible to correlate the antibacterial activity and the charge of the POT (Figure 3). The presented dependence may indicate for medium-sized POTs (but not for POTs with number of addenda atoms more than 22) a stronger effect against *M. catarrhalis* of anions exhibiting a charge of −8 to −6.

Cells of *M. catarrhalis* have on their surface low molecular weight lipopolysaccharides (LPS), also called lipooligosaccharides (LOS), which contribute to the increased hydrophobicity of its outer membrane and to the high susceptibility to hydrophobic antimicrobial agents such as macrolides (Gotoh et al., 1989; Tsujimoto et al., 1999). However, *M. catarrhalis* shows susceptibility not only to hydrophobic agents, but also to hydrophilic agents such as β-lactam antibiotics (Gotoh et al., 1992). The increased susceptibility of these strains toward β-lactams is probably due to the higher permeability of the outer membrane toward these agents. POMs, as examples of super chaotropic anions, can adsorb onto lipid monolayers via electrostatic and/or hydrophobic interaction depending on the charge of the lipid layer (Kobayashi et al., 2017). The model experiments with three differently charged Keggin anions show that dominant interaction equally depends both on the charge density of POMs and on the lipid density (Kobayashi et al., 2017).

### Time-killing studies

**Figure 4:**
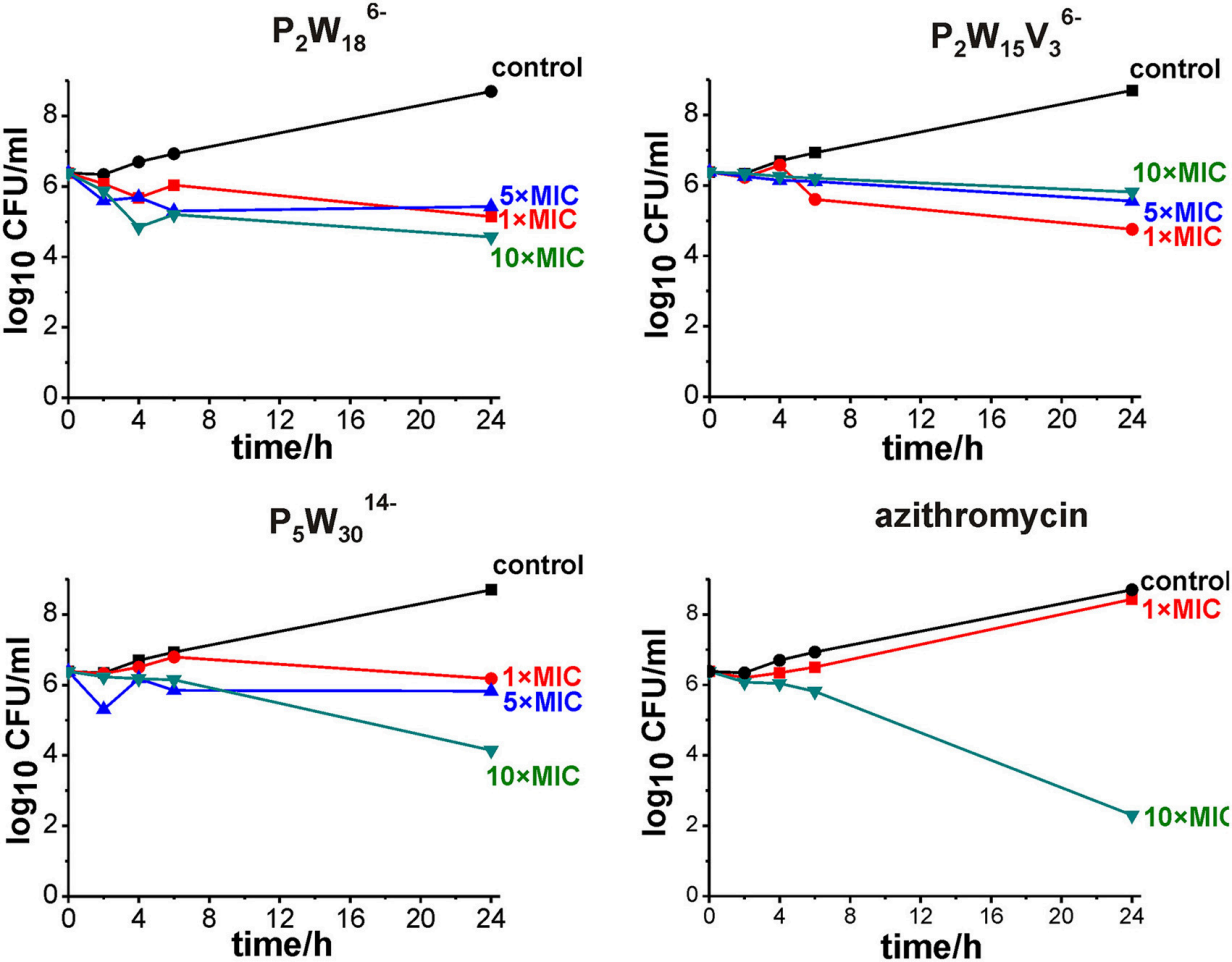
Time-kill curves for Dawson **P**_**2**_${\text{W}}_{18}^{6-}$ and **P**_**2**_**W**_**15**_${\text{V}}_{3}^{6-}$, Preyssler **P**_**5**_${\text{W}}_{30}^{14-}$ POTs and azithromycin at minimum inhibitory concentration (MIC) (red), 5-fold (blue) and 10-fold MIC concentration (green) against *M. catarrhalis* ATCC23246 strain. Control represents uninhibited bacterial growth (black).

In order to assess whether the tested compounds kill the bacteria (bactericidal effect) or prevent its growth (bacteriostatic effect), time-kill study was performed. Killing kinetics for three the most active compounds: Preyssler **P**_**5**_${\text{W}}_{30}^{14-}$ (Figure 1A) and two Dawson **P**_**2**_${\text{W}}_{18}^{6-}$ and **P**_**2**_**W**_**15**_${\text{V}}_{3}^{6-}$ (Figure 1B) POTs were determined against *M. catarrhalis*. POTs were tested at three concentrations, corresponding to 1×, 5×, and 10×MIC. The bactericidal activity of the agents was defined for at least a 3 log_10_ reduction in viable colony counts. In the control (sample without antibiotic), the numbers of the viable strain were kept within the cultivation of 24 h relative to those at 0 h. Figure 4 represents time-killing curves for compounds **P**_**5**_${\text{W}}_{30}^{14-}$, **P**_**2**_${\text{W}}_{18}^{6-}$, and **P**_**2**_**W**_**15**_${\text{V}}_{3}^{6-}$. All tested POMs show bacteriostatic effects, resulting from a little change in viable colony numbers within 24 h despite the concentration being equal to 10-fold MIC (Figure 4). Although it would seem preferable for an antibiotic to kill the offending bacteria rather than to merely inhibit it, the clinical importance of an *in vitro* bactericidal action being better than a bacteriostatic action has rarely been documented. The superiority of bactericidal over bacteriostatic action in the treatment of gram-positive bacterial infections is intuitive rather than based on rigorous scientific research (Pankey and Sabath, 2004).

## Conclusions

An important investigation in exploring biological effects of POMs was performed. The antibacterial activity of 29 POTs, POMos, and POVs against *M. catarrhalis* was investigated by determination of their minimum inhibitory concentrations (MIC) and time-killing kinetics. The following important conclusions were drawn:

1. According to their MIC values, Preyssler **P**_**5**_${\text{W}}_{30}^{14-}$ (Figure 1A) and five Dawson-based **P**_**2**_${\text{W}}_{18}^{6-}$, **P**_**2**_**W**_**15**_${\text{V}}_{3}^{6-}$, **P**_**2**_**O**_**7**_${\text{Mo}}_{18}^{4-}$, **As**_**2**_${\text{Mo}}_{18}^{6-}$, ${\text{AsW}}_{18}^{7-}$ (Figures 1B, 2) POMs are promising antibacterial agents against *M. catarrhalis*.
2. The Preyssler type POT **P**_**5**_${\text{W}}_{30}^{14-}$ (Figure 1A) showed the highest antibacterial activity against *M. catarrhalis* (MIC = 1 μg/ml) and further MIC investigation against *S. aureus* and *E. faecalis* proved its antibacterial potential.
3. Based on MIC values, Dawson-type POMs (see Figure 1B) exhibited highest activity and selectivity against *M. catarrhalis*.
4. Among Keggin-type POMs (see Figures 1C-E), only the mono-substituted ${\text{CoTiW}}_{11}^{6-}$ (Figure 1D) showed MIC comparable to that of the Dawson-type group.
5. POVs and Anderson type POMs (Figure 1I) were inactive (MIC > 256 μg/ml) against *M. catarrhalis* strain.
6. According to time-killing studies three the most active POTs (Preyssler **P**_**5**_${\text{W}}_{30}^{14-}$ and Dawson **P**_**2**_${\text{W}}_{18}^{6-}$ and **P**_**2**_**W**_**15**_${\text{V}}_{3}^{6-}$) showed bacteriostatic effect against *M. catarrhalis*.
7. POM activity mainly depends on composition, shape and size, but in the case of medium-size POTs correlates with the total net charge.

## Author contributions

NG and AR contributed toward the study design, wrote the manuscript. NG, EA-S, and LK synthesized and characterized POMs. HC-P and DV performed all antibacterial study. All authors read and approved the final version of the manuscript.

### Conflict of interest statement

The authors declare that the research was conducted in the absence of any commercial or financial relationships that could be construed as a potential conflict of interest.

## References

[B1] Bae E. Lee J. W. Hwang B. H. Yeo J. Yoon J. Cha H. J.. (2008). Photocatalytic bacterial inactivation by polyoxometalates. Chemosphere 72, 174-181. 10.1016/j.chemosphere.2008.01.071 18343481

[B2] Barry A. L. Craig W. A. Nadler H. Reller L. B. Sanders C. C. Swenson J. M. (1999). Methods for Determining Bactericidal Activity of Antimicrobial Agents: Approved Guideline. NCCLS document M26-A 19 (18). Wayne, PA: National Committee for Clinical Laboratory Standards.

[B3] Bi L. H. Wang E. B. Peng J. Huang R. D. Xu L. Hu C. W. (2000). Crystal structure and replacement reaction of coordinated water molecules of the heteropoly compounds of sandwich-type tungstoarsenates. Inorg. Chem. 39, 671-679. 10.1021/ic990596v 11272560

[B4] Bijelic A. Aureliano M. Rompel A. (2018). The antibacterial activity of polyoxometalates: structures, antibiotic effects and future perspectives. Chem. Commun. 54, 1153-1169. 10.1039/C7CC07549A 29355262 PMC5804480

[B5] Bijelic A. Rompel A. (2015). The use of polyoxometalates in protein crystallography-An attempt to widen a well-known bottleneck. Coord. Chem. Rev. 299, 22-38. 10.1016/j.ccr.2015.03.018 26339074 PMC4504029

[B6] Bijelic A. Rompel A. (2017). Ten good reasons for the use of the tellurium-centered Anderson-Evans polyoxotungstate in protein crystallography. Acc. Chem.Res. 50, 1441-1448. 10.1021/acs.accounts.7b00109 28562014 PMC5480232

[B7] Blazevic A. Rompel A. (2016). The Anderson-Evans polyoxometalate: from inorganic building blocks via hybrid organic-inorganic structures to tomorrows “Bio-POM”. Coord. Chem. Rev. 307, 42-64. 10.1016/j.ccr.2015.07.001

[B8] Briand L. E. Valle G. M. Thomas H. J. (2002). Stability of the phospho-molybdic Dawson-type ion P_2_Mo_18_${\text{O}}_{62}^{6-}$ in aqueous media. J. Mat. Chem. 12, 299-304. 10.1039/b106769a

[B9] Clemente-Juan J. M. Coronado E. Gaita-Ariño A. (2012). Magnetic polyoxometalates: from molecular magnetism to molecular spintronics and quantum computing. Chem. Soc. Rev. 41, 7464-7478. 10.1039/c2cs35205b 22948854

[B10] Contant R. Klemperer W. G. Yaghi O. (2007). Potassium octadecatungstodiphosphates(V) and related lacunary compounds, in Inorganic Syntheses, ed Ginsberg A. P. (New York, NY: John Wiley & Sons, Inc.), 104–111.

[B11] Del Beccaro M. A. Mendelman P. M. Inglis A. F. Richardson M. A. Duncan N. O. Clausen C. R.. (1992). Bacteriology of acute otitis media: a new perspective. J. Pediatr. 120, 81-84. 10.1016/S0022-3476(05)80605-5 1731029

[B12] Dizaj S. M. Lotfipour F. Barzegar-Jalali M. Zarrintan M. H. Adibkia K. (2014). Antimicrobial activity of the metals and metal oxide nanoparticles. Mater. Sci. Eng. C 44, 278-284. 10.1016/j.msec.2014.08.031 25280707

[B13] Evans H. T. Rollins O. W. (1976). Sodium paradodecatungstate 20-hydrate. Acta Cryst. B 32, 1565-1567. 10.1107/S0567740876005827

[B14] Finke R. G. Rapko B. Saxton R. J. Domaille P. J. (1986). Trisubstituted heteropolytungstates as soluble metal oxide analogs. 3^1^. synthesis, characterization, ^31^P, ^29^Si, ^51^V, and 1-and 2-D ^183^W NMR, deprotonation, and proton mobility studies of organic solvent solute forms of H_x_SiW_9_V_3_${\text{O}}_{40}^{\text{x}-7}$ and H_x_P_2_W_15_V_3_${\text{O}}_{62}^{\text{x}-9}$. J. Am. Chem. Soc. 108, 2947-2960. 10.1021/ja00271a025

[B15] Fu L. Gao H. Yan M. Shouzhu L. Xinyu L. Dai Z.. (2015). Polyoxometalate-based organic-inorganic hybrids as antitumor drugs. Small 11, 2938-2945. 10.1002/smll.201500232 25721026

[B16] Fukuda N. Yamase T. (1997). *In vitro* antibacterial activity of vanadate and vanadyl compounds against *Streptococcus pneumoniae*. Biol. Pharm. Bull. 20, 927-930. 10.1248/bpb.20.927 9300145

[B17] Gao J. Yan J. Beeg S. Long D. L. Cronin L. (2013). One-pot versus sequential reactions in the self-assembly of gigantic nanoscale polyoxotungstates. J. Am. Chem. Soc. 135, 1796-1805. 10.1021/ja309237x 23244039

[B18] Gao N. Sun H. Dong K. Ren J. Duan T. Xu C.. (2014). Transition-metal-substituted polyoxometalate derivatives as functional anti-amyloid agents for Alzheimer's disease. Nat. Commun. 5:3422. 10.1038/ncomms4422 24595206

[B19] Gotoh N. Tanaka S. Nishino T. (1989). Supersusceptibility to hydrophobic antimicrobial agents and cell surface hydrophobicity in *Branhamella catarrhalis*. FEMS Microbiol. Lett. 59, 211-213. 10.1111/j.1574-6968.1989.tb03112.x 2500380

[B20] Gotoh N. Tanaka S. Nishino T. (1992). Permeability of the outer membrane of *Moraxella catarrhalis* for β-lactam antibiotics. J. Antimicrob. Chemother. 29, 279-285. 10.1093/jac/29.3.279 1592697

[B21] Gumerova N. I. Melnik N. A. Rozantsev G. M. Baumer V. N. Radio S. V. (2015). Sodium heteropolyhexamolybdenumnickelate (II) Na_4_[Ni(OH)_6_Mo_6_O_18_]·16H_2_O with an Anderson anion: synthesis and crystal structure. J. Struct. Chem. 56, 926-933. 10.1134/S0022476615050157

[B22] Gumerova N. I. Roller A. Rompel A. (2016). [Ni(OH)_3_W_6_O_18_(OCH_2_)_3_CCH_2_OH]^4−^: the first tris-functionalized Anderson-type heteropolytungstate. Chem. Commun. 52, 9263-9266. 10.1039/C6CC04326G 27355393 PMC5040144

[B23] Hori T. Himeno S. (1987). Preparation of a yellow heteropoly molybdosulfate. Chem. Lett. 16, 53-56. 10.1246/cl.1987.53

[B24] Ichida H. Sasaki Y. (1983). The structure of hexaguanidinium octadecamolybdodiarsenate enneahydrate, (CH_6_N_3_)_6_[As_2_Mo_18_O_62_]·9H_2_O. Acta Cryst. C 39, 529-533. 10.1107/S0108270183005363

[B25] Inoue M. Segawa K. Matsunaga S. Matsumoto N. Oda M. Yamase T. (2005). Antibacterial activity of highly negative charged polyoxotungstates, K_27_[KAs_4_W_40_O_140_] and K_18_[KSb_9_W_21_O_86_], and Keggin-structural polyoxotungstates against *Helicobacter pylori*. J. Inorg. Biochem. 99, 1023-1031. 10.1016/j.jinorgbio.2005.01.010 15833325

[B26] Jeannin Y. Martin-Frere J. (1979). X-ray study of (NH_4_)_7_[H_2_AsW_18_O_60_]·16H_2_O: first example of a heteropolyanion containing protons and arsenic (III). Inorg. Chem. 18, 3010-3014. 10.1021/ic50201a013

[B27] Jeannin Y. Martin-Frere J. Choi D. J. Pope M. T. (2007). The sodium pentaphosphato(V)-triacontatungstate anion isolated as the ammonium salt, in Inorganic Syntheses, ed Ginsberg A. P. (New York, NY: John Wiley & Sons, Inc.), 115–118.

[B28] Judd D. A. Nettles J. H. Nevins N. Snyder J. P. Liotta D. C. Tang J.. (2001). Polyoxometalate HIV-1 protease inhibitors. A new mode of protease inhibition. J. Am. Chem. Soc. 123, 886-897. 10.1021/ja001809e 11456622

[B29] Karalus R. Campagnari A. (2000). *Moraxella catarrhalis*: a review of an important human mucosal pathogen. Microbes Infect. 2, 547-559. 10.1016/S1286-4579(00)00314-2 10865200

[B30] Klemperer W. G. (2007). Tetrabutylammonium isopolyoxometalates, in Inorganic Syntheses, ed Ginsberg A. P. (New York, NY: John Wiley & Sons, Inc.), 74–85.

[B31] Kobayashi D. Nakahara H. Shibata O. Unoura K. Nabika H. (2017). Interplay of hydrophobic and electrostatic interactions between polyoxometalates and lipid molecules. J. Phys. Chem. C 121, 12895-12902. 10.1021/acs.jpcc.7b01774

[B32] Komura A. Hayashi M. Imanaga H. (1976). Heteropolytungstates containing cobalt (II) or cobalt (III). Bull. Chem. Soc. Jpn. 49, 87-91. 10.1246/bcsj.49.87

[B33] Kortz U. Pope M. T. (1994). Polyoxometalate-diphosphate complexes. 2. structure of 18-molybdopyrophosphate, [(P_2_O_7_)Mo_18_O_54_]^4−^, which encloses a linear, eclipsed conformation of the pyrophosphate anion, and preliminary characterization of its one-and two-electron heteropoly blues. Inorg. Chem. 33, 5643-5646. 10.1021/ic00103a008

[B34] Kortz U. Savelieff M. G. Bassil B. S. Dickman M. H. (2001). A large, novel polyoxotungstate: [${\text{As}}_{6}^{\text{III}}$W_65_O_217_(H_2_O)_7_]^26−^. Angew. Chem. Int. Ed. 40, 3384-3386. 10.1002/1521-3773(20010917)40:18<3384::AID-ANIE3384>3.0.CO;2-O 11592145

[B35] Kraus W. Stephan H. Röllich A. Reck G. (2005). K_6_H_2_[CoTiW_11_O_40_]·13H_2_O, with a monotitanoundecatungstocobaltate (II) anion. Acta Cryst. E61, i35-i37. 10.1107/S1600536805005180

[B36] Lee U. Joo H. C. (2003). Potassium-sodium double salt of decavanadate, K_4_Na_2_[V_10_O_28_]·10H_2_O. Acta Cryst. E 59, i122-i124. 10.1107/S1600536803016453

[B37] Li J. Chen Z. Zhou M. Jing J. Li W. Wang Y.. (2016). Polyoxometalate-driven self-assembly of short peptides into multivalent nanofibers with enhanced antibacterial activity. Angew. Chem. Int. Ed. 55, 2592-2595. 10.1002/anie.201511276 26766581

[B38] Lode H. Borner K. Koeppe P. Schaberg T. (1996). Azithromycin - review of key chemical, pharmacokinetic and microbiological features. J. Antimicrob. Chemother. 37, 1-8. 8818841 10.1093/jac/37.suppl_c.1

[B39] López X. Fernández J. A. Poblet J. M. (2006). Redox properties of polyoxometalates: new insights on the anion charge effect. Dalton Trans. 1162–1167. 10.1039/B507599H 16482352

[B40] Miras H. N. Yan J. Long D. L. Cronin L. (2008). Structural evolution of “S”-shaped [H_4_W_22_O_74_]^12−^ and “§”-shaped [H_10_W_34_O_116_]^18−^ isopolyoxotungstate clusters. Angew. Chem. Int. Ed. 47, 8420-8423. 10.1002/anie.200802109 18825759

[B41] Molitor C. Bijelic A. Rompel A. (2017). The potential of hexatungstotellurate (VI) to induce a significant entropic gain during protein crystallization. IUCr J. 4, 34-740. 10.1107/S2052252517012349 29123675 PMC5668858

[B42] Müller C. E. Iqbal J. Baqi Y. Zimmermann H. Röllich A. Stephan H. (2006). Polyoxometalates-a new class of potent ecto-nucleoside triphosphate diphosphohydrolase (NTPDase) inhibitors. Bioorg. Med. Chem. Lett. 16, 5943-5947. 10.1016/j.bmcl.2006.09.003 16997558

[B43] Nomiya K. Torii H. Hasegawa T. Nemoto Y. Nomura K. Hashino K.. (2001). Insulin mimetic effect of a tungstate cluster. Effect of oral administration of homo-polyoxotungstates and vanadium-substituted polyoxotungstates on blood glucose level of STZ mice. J. Inorg. Biochem. 86, 657-667. 10.1016/S0162-0134(01)00233-1 11583783

[B44] Pankey G. A. Sabath L. D. (2004). Clinical relevance of bacteriostatic versus bactericidal mechanisms of action in the treatment of Gram-positive bacterial infections. Clin. Infect. Dis. 38, 864-870. 10.1086/381972 14999632

[B45] Phillips M. A. (1950). The preparation of phosphotungstic acid and of sodium and barium phosphotungstates. J. Chem. Technol. Biotechnol. 69, 282-284. 10.1002/jctb.5000690906

[B46] Pope M. (1983). Heteropoly and Isopoly Oxometalates, Inorganic Chemistry Concepts. Berlin: Springer.

[B47] Proust A. Thouvenot R. Gouzerh P. (2008). Functionalization of polyoxometalates: towards advanced applications in catalysis and materials science. Chem. Commun. 1837–1852. 10.1039/B715502F 18401495

[B48] Rozantsev G. M. Radio S. V. Gumerova N. I. Baumer V. N. Shishkin O. B. (2009). Phase formation in the Ni^2+^-W${\text{O}}_{4}^{2-}$-H^+^-H_2_O system (Z= 1.00). crystal structure and properties of sodium heteropolyhexatunsten nickelate (2+) Na_4_[Ni(OH)_6_W_6_O_18_]·16H_2_O. J. Struct. Chem. 50, 296-305. 10.1007/s10947-009-0041-z

[B49] Sadakane M. Steckhan E. (1998). Electrochemical properties of polyoxometalates as electrocatalysts. Chem. Rev. 98, 1, 219-238. 10.1021/cr960403a 11851504

[B50] Saha A. K. Crans D. C. Pope T. M. Simone C. M. Glew R. H. (1991). Inhibition of human seminal fluid and leishmania donovani phosphatases by molybdate heteropolyanions. J. Biol. Chem. 266, 3511-3517. 1995614

[B51] Schmidt K. J. Schrobilgen G. J. Sawyer J. F. (1986). Hexasodium hexatungstotellurate (VI) 22-hydrate. Acta Cryst. C42, 1115-1118. 10.1107/S0108270186093204

[B52] Schwendt P. Tracey A. S. Tatiersky J. Gáliková J. Žák Z. (2007). Vanadium(V) tartrato complexes: speciation in the H_3_O^+^(OH^−^)/H_2_V${\text{O}}_{4}^{-}$/(*2R,3R*)-tartrate system and X-ray crystal structures of Na_4_[V_4_O_8_(rac-tart)_2_]·12H_2_O and (NEt_4_)_4_[V_4_O_8_((R,R)-tart)_2_]·6H_2_O (tart = C_4_H_2_${\text{O}}_{6}^{4-}$). Inorg. Chem. 46, 3971-3983. 10.1021/ic062223h 17419617

[B53] Shah H. S. Al-Oweini R. Haider A. Kortz U. Iqbal J. (2014). Cytotoxicity and enzyme inhibition studies of polyoxometalates and their chitosan nanoassemblies. Toxicol. Rep. 1, 341-352. 10.1016/j.toxrep.2014.06.001 28962250 PMC5598103

[B54] Tajima Y. (2001). Lacunary-substituted undecatungstosilicates sensitize methicillin-resistant *Staphylococcus aureus* to β-lactams. Biol. Pharm. Bull. 24, 1079-1084. 10.1248/bpb.24.1079 11558574

[B55] Téazéa A. Hervéa G. Finke R. G. Lyon D. K. (2007). α-, β-, and γ-dodecatungstosilicic acids: isomers and related lacunary compounds, in Inorganic Syntheses, ed Ginsberg A. P. (New York, NY: John Wiley & Sons, Inc.), 85–96.

[B56] Tourné C. Revel A. Tourné G. Vendrell M. (1973). Heteropolytungstates containing elements of phosphorus family with degree of oxidation (III) or (V)-identification of species having composition X_2_W_19_ and XW_9_ (X = P, As, Sb, Bi) and relation to those with composition XW_11_. C. R. Acad. Sci. Ser. C277, 643-645.

[B57] Tsujimoto H. Gotoh N. Nishino T. (1999). Diffusion of macrolide antibiotics through the outer membrane of *Moraxella catarrhalis*. J. Infect. Chemother. 5, 196-200. 10.1007/s101560050034 11810516

[B58] Wang S. S. Yang G. Y. (2015). Recent advances in polyoxometalate-catalyzed reactions. Chem. Rev. 115, 4893-4962. 10.1021/cr500390v 25965251

[B59] Wikler M. A. (2009). Methods for Dilution Antimicrobial Susceptibility Test for Bacteria That Grow Aerobically. Approved Standard M7-A8. Wayne, PA: Clinical and Laboratory Standards Institute.

[B60] Yamase T. (2005). Anti-tumor,-viral, and -bacterial activities of polyoxometalates for realizing an inorganic drug. J. Mater. Chem. 15, 4773-4782. 10.1039/b504585a

[B61] Yamase T. Fukuda N. Tajima Y. (1996). Synergistic effect of polyoxotungstates in combination with β-lactam antibiotics on antibacterial activity against methicillin-resistant *Staphylococcus aureus*. Biol. Pharm. Bull. 19, 459-465. 10.1248/bpb.19.459 8924919

[B62] Yamase T. Pope M. (2006). Polyoxometalate Chemistry for Nano-Composite Design. New York, NY: Springer Science & Business Media.

